# Tianzhi granule improves cognition and BPSD of vascular dementia: a randomized controlled trial

**DOI:** 10.1186/s12967-020-02232-z

**Published:** 2020-02-13

**Authors:** Jing Shi, Mingqing Wei, Jingnian Ni, Feng Sun, Li Sun, Junfu Wang, Tao Yu, Kai Wang, Peiyuan Lv, Yunfu Wang, Yulian Zhang, Xuguang Gao, Xuanzhao Gao, Benyan Luo, Shanping Mao, Baorong Zhang, Xiangyang Ren, Fengchun Yu, Wenli Hu, Ping Yin, Nanjin Wu, Xianfeng Liu, Qi Bi, Yongyan Wang, Jinzhou Tian, The CHASE Study Group

**Affiliations:** 1 Department of Neurology, Dongzhimen Hospital, Beijing University of Chinese Medicine, Beijing, 100007 China; 2 Zhongjing Wanxi Pharmaceutical Co., Ltd., Nanyang, 474550 Henan Province China; 3 Department of Neurology, First Hospital of Jilin University, Changchun, 130031 Jilin Province China; 4 Department of Acupuncture, Kaifeng Hospital of Traditional Chinese Medicine, Kaifeng, 475000 Henan Province China; 5 Department of Acupuncture, First Teaching Hospital of Tianjin University of Traditional Chinese Medicine, Tianjin, 300193 China; 6 Department of Neurology, First Affiliated Hospital of Anhui Medical University, Hefei, 230022 Anhui Province China; 7 Department of Neurology, Hebei General Hospital, Shijiazhuang, 050051 Hebei Province China; 8 Department of Neurology, Taihe Hospital, Hubei University of Chinese Medicine, Shiyan, 442000 Hubei Province China; 9 Department of Neurology, Second Affiliated Hospital of Tianjin University of Traditional Chinese Medicine, Tianjin, 300250 China; 10 Department of Neurology, Peking University People’s Hospital, Beijing, 100044 China; 11 Department of Neurology, Xinxiang Central Hospital, Xinxiang, 453700 Henan Province China; 12 Department of Neurology, First Affiliated Hospital, Zhejiang University School of Medicine, Hangzhou, 310003 Zhejiang Province China; 13 Department of Neurology, Renmin Hospital of Wuhan University, Wuhan, 430072 Hubei Province China; 14 Department of Neurology, Second Affiliated Hospital of Zhejiang University, Hangzhou, 310009 Zhejiang Province China; 15 Department of Neurology, Luoyang Central Hospital Affiliated to Zhengzhou University, Luoyang, 471009 Henan Province China; 16 Department of Neurology, Beijing Haidian Hospital, Beijing, 100080 China; 17 Department of Neurology, Beijing Chao-Yang Hospital Affiliated to Capital Medical University, Beijing, 100020 China; 18 Department of Epidemiology and Biostatistics, School of Public Health, Tongji Medical College, Huazhong University of Science and Technology, Wuhan, 430030 Hubei Province China; 19 Department of Neurology, Beijing Anzhen Hospital, Capital Medical University, Beijing, 100029 China; 20 Institute of Basic Research in Clinical Medicine, China Academy of Chinese Medical Sciences, Beijing, 100700 China

**Keywords:** Vascular dementia, Tianzhi granule, Herbal medicine, Randomized controlled trial, Behavioral and psychological symptoms of dementia

## Abstract

**Background and purpose:**

Tianzhi granule (TZ) is usually used for patients with vascular dementia (VaD) in China. The aim was to assess the effect of TZ by a randomized clinical trial (RCT).

**Methods:**

A 24-week RCT was conducted in 16 centres. Participants were grouped into TZ, donepezil or placebo. The co-primary outcomes were the Vascular Dementia Assessment Scale-cognitive subscale (VADAS-cog) and Clinician’s Interview-based Impression of Change-plus caregiver information (CIBIC-plus).

**Results:**

A total of 543 patients with mild to moderate VaD were enrolled, of whom 242 took TZ granules, 241 took donepezil, and 60 took placebo. The least-squares mean changes from baseline and 95% CI were 6.20 (5.31, 7.09) (TZ group), 6.53 (5.63, 7.42) (donepezil group) and 3.47 (1.76, 5.19) (placebo group), both TZ and donepezil showed small but significantly improvement compared with placebo group. The percent of improvement on the global impression which was measured by CIBIC-plus was 73.71% in TZ and 58.18% in placebo, there was significant different between TZ and placebo group (P = 0.004). No significant differences were observed between TZ and donepezil. No significant differences of adverse events were found.

**Conclusions:**

TZ and donepezil could bring symptomatic benefit for mild to moderate VaD.

*Trial registration* The protocol had retrospectively registered at clinical trial.gov, Unique identifier: NCT02453932, date of registration: May 27, 2015; https://www.clinicaltrials.gov/ct2/show/NCT02453932?term=NCT02453932&rank=1

## Background

Vascular dementia (VaD) is the second most common cause of dementia after Alzheimer’s disease (AD) [1], and its prevalence is higher than excepted [2, 3]. The prevalence of post-stroke dementia was found to be up to 32%, four to six times higher than in individuals free of stroke [4]. A few of epidemiological studies on VaD have been done in China, but the estimates of the prevalence and incidence remain inconsistent because of the use of different sampling methods. A meta-analysis showed that the prevalence in a population aged 60 years or older for VaD was 0.9% [5]. It was estimined that patients with VaD constitute the second largest population of people with dementia in China (2•49 million people aged 65 years and older) [5, 6]. Because dementia can showed a decline in patients’ cognition and ability of daily living, which lead to incapable of their own work and need the care of others. As aging demographic transition is proceeding rapidly especially in China, dementia is rapidly becoming the major public health problem.

Several investigators have tried to evaluate the effect of cholinesterase inhibitors and memantine which respectively produces small benefits in cognition in patients with mild to moderate VaD, but no behavioral or functional benefits were observed [7]. No drugs have been approved for the treatment of VaD until now. In fact, 92% of patients with VaD exhibited one or more abnormal behaviors associated with dementia [8]. Behavioral and psychological symptoms of dementia (BPSD) are predictors of care burden and psychological distress [9], and may jeopardize safety or promote institutionalization [10, 11]. Atypical antipsychotic medications have been commoly used to treat BPSD. However, such medications were associated with worsening cognitive function over 36 weeks’ treatment, including Mini-mental State Examination (− 2.4 points) and Alzheimer’s Disease Assessment Scale-cog (+ 4.4 points), which was consistent with 1 year’s deterioration compared with placebo [12]. Besides, the antipsychotics were also associated with increased mortality in older adults with dementia [13].

In the Traditional Chinese Medicine, dementia, especially the neuropsychiatric symptoms, such as irritability, agitation, and anxiety were correlated to “liver fire”, as results of imbalance between yin and yang of liver function, and the treatment method was calming the liver and restraining the Yang to reduce liver fire. Tianzhi granule (TZ), a traditional herbal drug, attempt to fulfill to this need. TZ has been approved by the China Food and Drug Administration (CFDA) for the treatment of VaD (batch number: Z20040041). Studies have showed that Tianzhi granule could inhibit the proliferation of astroglial cells by promoting pre-nerve cells proliferation to improve the learning and memory ability of vascular dementia rats [14]. Several studies were conducted to evaluate the efficacy of TZ for mild to moderate VaD [15]. A meta-analysis has indicated that Tianzhi granule is safe and effective medicine for treating VaD [16]. However, there were some flaws in design of the previous trials, such as absence of placebo control group, relatively small sample size, short follow-up period and inappropriate outcome measure. This study aimed to further investigate the effects of TZ granule on the cognition and BPSD in mild to moderate VaD patients of the Chinese Alzheimer’s disease Study and Evaluation project (CHASE).

## Methods

### Study design

This study was a phase III clinical trial designed as a randomized, double-blind, parallel, three arms, multi-centre study.

### Participants

#### Inclusion criteria

Patients, aged ≥ 45 and ≤ 85 years old, Chinese speaking in both gender meeting a diagnosis of possible or probable VaD > 6 months’ duration, according to the National Institute of Neurological Disorders and Stroke–Association Internationale pour la Recherche et l’Enseignement en Neurosciences (NINDS-AIREN) criteria were enrolled [17]. The diagnosis of VaD took into account clinical and imaging evidence of cerebrovascular diseases, evidence of ischemic stroke on MRI, including infarct in the main blood vessels, single strategic infarct (e.g., thalamus, angular gyrus, and basal forebrain), multiple lacunar infarcts, and/or extensive white matter damage surrounding ventricles (≥ 25% of all white matter area). Other inclusion criterias were as follows: (1) the severity of dementia assessed as mild to moderate was defined by a score of 14 to 26 on the Mini-mental state examination (MMSE) [18]; (2) the subjects were also required to adequate vision and hearing to participate in study assessments; (3) weighting between 45 and 90 kg; (4) Hachinski ischemic scale (HIS) > 7 [19]; (5) with a stable caregiver.

#### Exclusion criteria

Exclusion criteria were as follows: a medical history of other dementia types, like Alzheimer’s disease (MRI showed significant medial temporal lobe atrophy adjusted age), Parkinson’s disease dementia, Huntington disease, Normal pressure hydrocephalus, et al.; major depression (the Hamilton Depression Scale (HAMD for 17 items > 17) or psychotic disorder [20]; acute stage of cerebral hemorrhage or subarachnoid hemorrhage; hypothyroidism; drug or alcohol abuse; epilepsy history; myasthenia gravis history; severe cardiovascular disease (severe arrhythmia with heart rate ≥ 100 or ≤ 60 times per min, leftbundlebranch block, myocardial infarction within 3 months, systolic pressure ≥ 180 mmHg or ≤ 90 mmHg); severe liver or kidney dysfunction (alanine aminotransferase > 60 IU/L, aspartate transaminase > 60 IU/L or serumcreatinine > 266 μmol/L); severe asthma or chronic obstructive pulmonary disease; gastrointestinal tract obstruction or severe peptic ulcer; glaucoma; administration of cholinesterase inhibitors, memantine or nimodipine in the last month; use of sympathomimetic agent, antihistamine drug, anti-anxiety drugs or tranquilizer within 48 h before assessment; use of antipsychotic drugs within 72 h before assessment; participation in other clinical trials; allergic history to any type of medication used in this study.

The Ethics Committee of the Beijing Anzhen Hospital, Capital medical university, has approved this study, also approved by each center where the study conducted. The patients and responsible caregivers were asked to provide written informed consent. The study was conducted according to Good Clinical Practice Guidelines and the principles of the Declaration of Helsinki. This study had registered at Clinical trial gov at May 27, 2015, unique identifier: NCT02453932, the website was Clinical Trial Registration-URL: https://clinicaltrials.gov/ct2/show/NCT02453932?term=Tianzhi&rank=1. The protocol was failed to register before participant recruitment. The registration name was “Efficacy and Safety of Tianzhi Granule in Mild to Moderate Vascular Dementia”.

### Study medication

In the 2 weeks placebo run-in period, all patients received the placebo identified to TZ (5 g, 3 times per day) and placebo identified to donepezil. During the double-blind 24 weeks’ medication, the patients were randomly allocated to 3 groups: (1) TZ group (1 pack TZ (5 g), 3 times per day and placebo identified to donepezil); (2) donepezil group (donepezil 5 mg per day and placebo identified to TZ (5 g, 3 times per day)); (3) placebo group (placebo identified to TZ and placebo identified to donepezil). Both TZ and placebo (batch number:20121201) were produced by Zhongjing Wanxi Pharmaceutical Co., Ltd (national medicine approval number: Z20040041). Donepezil (Aricept) were produced by Eisai China Inc (national medicine approval number: H20040020), and repacked by Zhongjing Wanxi Pharmaceutical Co., Ltd with external package (repacked batch number:20121201) identified to placebo. To preserve blinding, the placebo had an identical taste and appearance to the experimental drugs.

TZ, an approved Chinese herbal medicine by China FDA for VaD, derived from ancient herbal prescription Tianma-Gouteng-Yin which is composed of 12 herbs, including Rhizoma Gastrodiae (tiān má), Ramulus Uncariae Cum Uncis (gōu téng), Concha Haliotidis (shí jué míng), Cortex Eucommiae (dù zhòng), Herba Taxilli (sāng jì shēng), Caulis Polygoni Multiflori (shŏu wū téng), Sclerotium Poriae Pararadicis (fú shén), Fructus Gardeniae (zhī zĭ), Flos Sophorae (huái huā), Radix Scutellariae (huáng qín), Herba Leonuri (yì mŭ căo) and Radix Cyathulae (chuān niú xī). The main active ingredients of TZ include gastrodin, geniposide, rutin, baicalinand so on. All subjects received a sicmilar dose of the active ingredients.

### Efficacy measurements

#### Primary efficacy assessment

The changes of the Vascular Dementia Assessment Scale-Cognitive Subscale (VADAS-cog/17items) from baseline after 24 weeks treatment were adopted as the primary endpoint [21]. VADAS-cog is a revision of the ADAS-cog to be a better measure in vascular conditions [22]. In addition to items in the ADAS-cog, the VADAS-cog includes additional frontal lobe tests reflecting attention, working memory, executive function, and verbal fluency [16] It was suggested that the VADAS-cog may be a more sensitive endpoint than ADAS-cog in studies of patients with white matter load and vascular burden of the brain [23].

The other primary efficacy measurement was the change of Clinician Interview-Based studies Impression of Change scale-plus version (CIBIC-plus). The CIBIC-plus is a 7-point scale which provides an index of clinically important change for dementia patients. It is a global measure of detectable change in concentration, orientation, memory, language, behavior, initiative and activities of daily living, usually requiring separate interviews with patients and caregivers [24]. The score of CIBIC-plus ranges from 1 to 7, and the score of 1–3 indicates improvement, 4 means no changed, and 5–7 indicates worse.

### Secondary efficacy assessment

The Neuropsychiatric inventory (NPI) was used to assess changes in 12 types behavioral disturbances occurring in dementia patients [25]. MMSE was used to evaluate global cognition. Executive function was assessed by clock drawing test (4 points) [26], and Trail making test part A and B test [27]. Activity of daily living scale was used to measure the physical self-maintenance ability and instrumental activities of daily living ability [28]. Score changes of the above scale were secondary efficacy indicators.

### Safety assessment

The safety assessments included the following: (1) physical examination of vital signs, including rate of breathing, heart rate, and blood pressure; (2) electrocardiography; (3) The laboratory parameters included complete blood count, urine routine test, fecal routine and occult blood test, hepatic and renal function, coagulation function and electrocardiogram (ECG) and (4) any adverse events that may occur, including the types of adverse events, time of occurrence, duration, treatment measures, and evaluation of the correlation between the tested drugs and the adverse event (positive, probable, possible, or not correlated); the severity of the adverse event (mild, moderate, and severe) must be evaluated.

### Procedure

This study included a 2-week run-in period, and followed a 24-week double blind treatment. Efficacy measurements were taken at baseline, and at weeks 4, 12 and 24. And the safety measurements were conducted at the baseline, 12th and 24th week. During these visits, neuropsychological evaluations, physical and neurologic examinations, laboratory determinations, vital signs measurements, medication compliance checks, and AE monitoring were performed.

### Sample size calculations

On the primary efficacy measurement, the decrease in VADAS-cog score after treatment, a superiority test between the treatment and placebo control groups was performed. Based on the current literature and with reference to expert discussions, the expected difference of the mean decreases in VADAS-cog scores in the treated group, compared with in the placebo control group, is approximately 3.5 (Δ = μt − μr) [29], the common variance σ^2^ = 36.0. Set α = 0.05 (two sides), β = 0.20, power = 0.80 and the ratio of the treatment group to the placebo group is 4:1. The number of cases, from a statistical perspective, is no less than 136 in the treatment group and 34 in the placebo group.

A non-inferiority test between the TZ and donepezil groups was performed. The non-inferiority value was − 1.6.

Taking into account the factor of falling-off, an approximately 10% increase in cases is necessary. Finally, a total sample size of 540 cases is determined as sufficient for this clinical trial. Among these, 240 cases are in the treatment group, 240 in the positive control group and 60 in the placebo control group.

By a block randomization, 9 qualified subjects in each block are randomly assigned to the treatment, positive control and placebo groups at a ratio of 4:4:1.

### Randomization and masking

In this trial, a complete randomization was adopted. Eligible subjects were randomly assigned to groups A, B or C. The specific method is as follows: the numbers of the observed cases were each labeled “No.XXX”. Using SAS 9.3, a biostatistics expert developed a computing procedure statement with set seeds, and a random number table was generated. Based on the table, a series of random numbers emerged and these were matched individually with each case number. Subjects were divided into groups A (Tianzhi group), B (Donepezil group) or C (Placebo group) at the ratio of 4:4:1. According to the case number and grouping, each subject was provided with the appropriate kit, with a drug number matching the case number. Based on the case number, random number and grouping, emergency sealed envelopes were prepared and sent to the hospitals involved in the trial. The outside of each envelope was marked with the case number. When a qualified subject was enrolled into the trial, the kit with the appropriate case number, in the order of subject enrollment, was provided. The first blinding was case number matching groups, i.e., group A, B or C. The second blinding was the disposal among the three groups. The blinding was sealed separately, duplicated, and stored in the research unit and the pharmaceutical factory. Once the blinding was broken, the patient were managed as off-trial. Patients, caregivers, the study investigator, any other personnel involved in the study, and the investigating staffs of the Henan Wanxi pharmaceutical Co were blinded until all patients complete the study and all data were collected.

### Statistics

The statistical analyses were conducted in three populations. The full analysis set (FAS), which based on the intent-to-treat population (ITT), consisting of all randomized population who toke at least one dose of medication and at least one primary efficacy evaluation on treatment. The per protocol set (PPS) included all randomized patients who had received at least 80% assigned 24 weeks’ double-blind medication with complete record of efficacy variable, with no major protocol violations. Safety and tolerability were assessed for all randomized patients who received the study medication (safety set, SS). AEs were considered as serious (SAEs) according to standard criteria. For missing data at endpoint, a last observation carried forward (LOCF) analysis was used. ANCOVA models that included baseline score, treatment, and centre as covariates were used to assess differences between the treatment groups for linear efficacy measures. Two primary analyses were conducted, one comparing the TZ and placebo groups using superiority test, and one comparing the TZ and donepezil groups using non-inferiority analysis. Categorical efficacy assessments were analyzed with a Cochran-Mantel–Haenszel test. The least squares mean changes from baseline scores to endpoint were presented for variables analyzed with the ANCOVA models.

The numbers needed to treat (NNTs) were based on the proportion (p) of responders (patients improved on CIBIC-plus) for each treatment group (P_Placebo_, P_TZ_, P_donepezil_). Briefly, the NNT formula used for TZ and donepezil treatment groups was:

$${\text{TZ group}}{:}{\text{ NNT}} = 1/\left( {{\text{P}}_{\text{TZ}} - {\text{ P}}_{\text{Placebo}} } \right)$$

$${\text{Donepezil group}}{:}{\text{ NNT}} = 1/\left( {{\text{P}}_{\text{donepezil}} - {\text{ P}}_{\text{Placebo}} } \right)$$

## Results

**Fig. 1:**
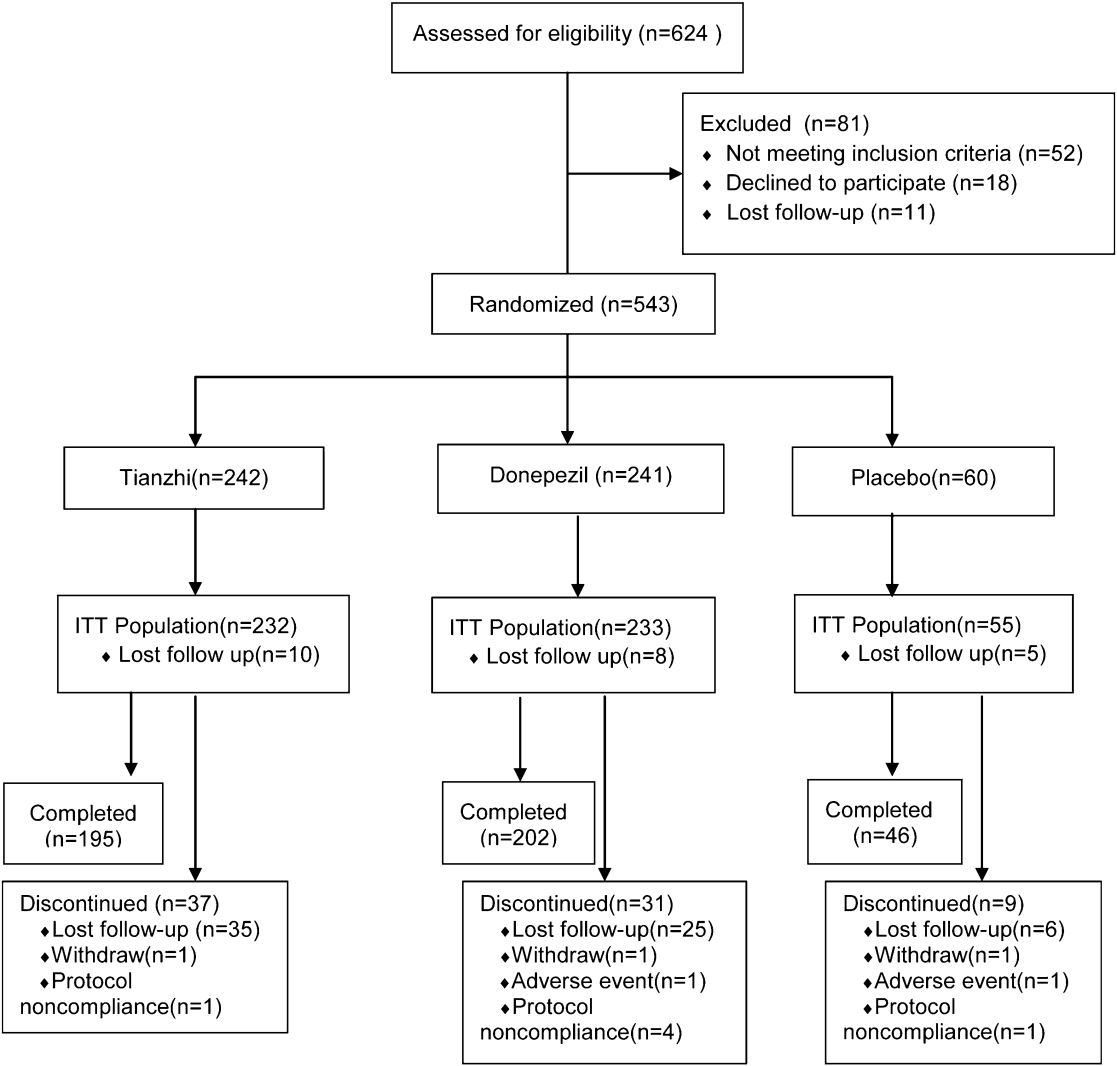
The study flow diagram. *ITT* Intent-to-treat

Because 7 study centres have not enrolled participant, 16 centres conducted this study finally. A total of 624 subjects were screened and 543 entered the study and were randomized at last from October 2013 to May 2017. 242 patients were assigned to receive TZ, 241 were randomized to donepezil group, and 60 in the placebo. 10 patients in TZ, 8 in donepezil and 5 in placebo were did not take medication and lost to follow-up after randomization, and 520 patients entered the ITT population finally (232 in TZ, 233 in donepezil, 55 in placebo). Of these 520 patients, 75 subjects discontinued their treatments, and reasons for discontinued medication were shown in Fig. 1. The overall completion rate of this study was 81.6%, and there was no significant difference between three treatment groups in completion rates (placebo, 76.67%; donepezil, 83.82%; TZ, 80.58%). All 520 patients were included in the safety analyses. Demographic characteristics of patients in all groups were similar at baseline (Table 1).

**Table 1:** Patient demographics and baseline characteristics

Items	Tianzhi (n = 232)	Donepezil (n = 233)	Placebo (n = 55)
Gender, male/female	154/78	149/84	35/20
Race, han/other	226/6	233/0	54/1
Age, mean (SD)	64.72 (9.18)	64.31 (9.99)	63.95 (9.15)
Education			
Primary school, N (%)	82 (35.34)	85 (36.48)	23 (41.82)
Middle school and above, N (%)	150 (64.66)	148 (63.52)	32 (58.18)
Smoking history, (yes/no)	97/134	96/136	24/31
Drinking history, (yes/no)	80/151	69/163	18/37
Neuropsychological score, Mean (SD)			
VADAS-cog	52.64 (10.59)	53.29 (10.17)	52.22 (11.36)
MMSE	20.56 (3.36)	20.56 (3.24)	20.51 (2.97)
TMT-A	112.05 (41.03)	113.26 (45.52)	124.69 (47.46)
TMT-B	208.42 (87.42)	209.42 (88.92)	235.83 (71.05)
ADL	15.15 (9.62)	14.66 (9.76)	14.87 (8.81)
CDT	2.48 (1.21)	2.53 (1.20)	2.35 (1.27)
NPI	5.31 (5.52)	5.35 (4.91)	5.40 (5.51)

*VADAS-cog* vascular dementia assessment scale-cognitive subscale, *NPI* Neuropsychiatric Inventory, *MMSE* mini-mental state examination, *ADL* activity of daily living scale, *CDT* clock drawing test, *TMT-A* trail making test part A, *TMT-B* trail making test part B

At least one concomitant medication was used by 356/520 patients (67.9%) during the study. There were no differences in concomitant medication usage among treatment groups (placebo, 65.45%, donepezil, 67.8%, and TZ, 70.26%).

### Primary efficacy outcomes

#### VADAS-cog

**Fig. 2:**
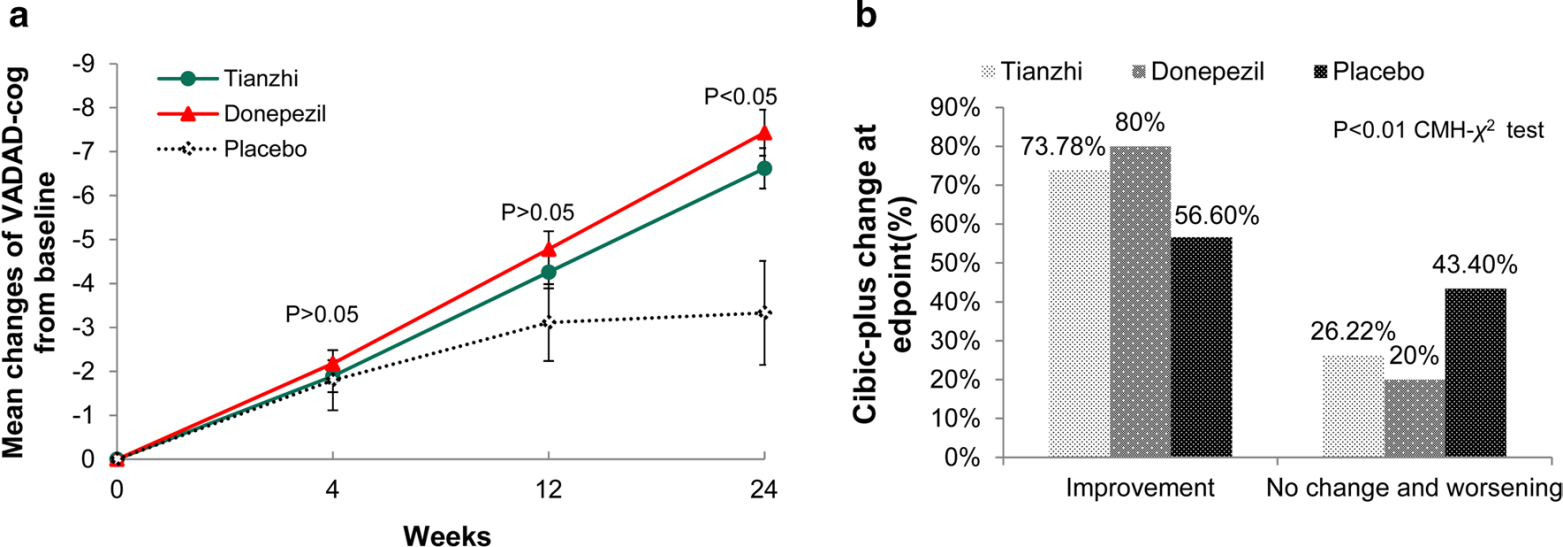
The primary efficacy measures at endpoint (ITT-LOCF analysis). **a** VADAS-cog mean change from baseline score in three groups. *VADAS-cog* Vascular Dementia Assessment Scale-Cognitive Subscale (VADAS-cog). p < 0.05 Tianzhi vs placebo in the mean change after 24 weeks’ treatment, p < 0.05 donepezil vs placebo in the mean change after 24 weeks’ treatment, p > 0.05 Tianzhi vs donepezil. *ITT* intent-to-treat, *LOCF* last observation carried forward, *PPS* per protocol set. **b** CIBIC-plus at endpoint (week 24) in three groups. *CIBIC-plus* Clinician Interview-Based Impression of Change scale Clinician’s Interview-Based Impression of Change–plus version. Overall treatment p < 0.01. p < 0.01 Tianzhi vs placebo, p < 0.01 donepezil vs placebo, p > 0.05 donepezil vs Tianzhi

Both patients treated with donepezil and TZ showed significant improvement compared with those taking placebo on the VADAS-cog at end point at week 24 (Fig. 2a) (Table 2).

**Table 2:** Efficacy measure outcomes (Neuropsychological score changes) in three groups at week 24 ITT-LOCF and week 24 PPS population

	Week 24 LOCF				Week 24 PPS			
	Tianzhi (n = 232)	Donepezil (n = 233)	Placebo (n = 55)	*p*	Tianzhi (n = 195)	Donepezil (n = 202)	Placebo (n = 46)	*p*
VADAS-cog, mean (SD)	− 6.12 (6.91)	− 6.72 (7.95)	− 3.47 (8.26)	0.004	− 6.66 (6.96)	− 7.40 (8.21)	− 3.70 (8.60)	0.001
CIBIC-plus								
Improvement, N (%)	171 (73.71)	186 (79.82)	32 (58.19)	0.004	155 (79.48)	170 (84.13)	27 (58.7)	< 0.001
Stable, N (%)	51 (21.98)	45 (19.31)	11 (20.00)		31 (15.90)	30 (14.85)	8 (17.39)	
Deterioration, N (%)	10 (4.31)	2 (0.86)	12 (21.82)		9 (4.61)	2 (0.99)	11 (23.91)	
NPI, mean (SD)	− 3.03 (4.84)	− 2.21 (5.45)	− 0.36 (5.70)	0.019	− 2.96 (4.78)	− 2.27 (5.45)	− 0.27 (5.52)	0.016
MMSE, mean (SD)	2.19 (2.52)	2.61 (2.60)	1.35 (2.67)	0.025	2.19 (2.53)	2.62 (2.61)	1.32 (2.69)	0.023
TMT-A, mean (SD)	− 12.86 (30.80)	− 20.60 (34.97)	− 11.92 (37.38)	0.009	− 12.97 (30.85)	− 20.76 (35.11)	− 12.94 (37.86)	0.015
TMT-B, mean (SD)	− 28.91 (63.96)	− 33.96 (70.53)	− 34.26 (63.08)	0.439	− 29.18 (64.02)	− 33.92 (69.13)	− 35.04 (63.59)	0.523
ADL, mean (SD)	− 1.84 (5.42)	− 1.84 (6.03)	− 0.96 (6.32)	0.859	− 1.87 (5.41)	− 1.88 (6.04)	− 1.00 (6.33)	0.856
CDT, mean (SD)	0.46 (1.10)	0.58 (1.15)	0.40 (1.18)	0.411	0.46 (1.11)	0.59 (1.15)	0.47 (1.14)	0.521

*VADAS-cog* vascular dementia assessment scale-cognitive subscale, *CIBIC-plus* clinician’s interview-based impression of change-plus care interview, *NPI* Neuropsychiatric Inventory, *MMSE* mini-mental state examination, *ADL* activity of daily living scale, *CDT* clock drawing test, *TMT-A* trail making test part A, *TMT-B* trail making test part B, *ITT* intent-to-treat, *LOCF* last observation carried forward, *PPS* per protocol set

The least-squares mean changes from baseline and 95% CI were 6.20 (5.31, 7.09) (TZ group), 6.53 (5.63, 7.42) (donepezil group) and 3.47 (1.76, 5.19) (placebo group). The difference between TZ and donepezil was − 0.33 (− 1.47, 0.82).

The difference between TZ and placebo was 2.73 (0.88, 4.58), and 3.05 (1.20, 4.91) between donepezil and placebo, both TZ and donepezil showed small but significantly improvement compared with placebo group.

#### CIBIC-Plus

In the ITT population, the improvement rates on CIBIC-plus of the TZ group (n = 171, 73.71%) and the donepezil group (n = 186, 79.82%) were significantly higher than that of the placebo group (n = 32, 58.19%) (p < 0.001) (Fig. 2b, Table 2). Compared with the placebo group at endpoint, significant improvements on the CIBIC-plus were observed in TZ and donepezil treatment groups (TZ treatment, p = 0.005; donepezil, p = 0.008). The same results were obtained in the PPS population.

### Secondary efficacy outcomes

#### NPI

**Fig. 3:**
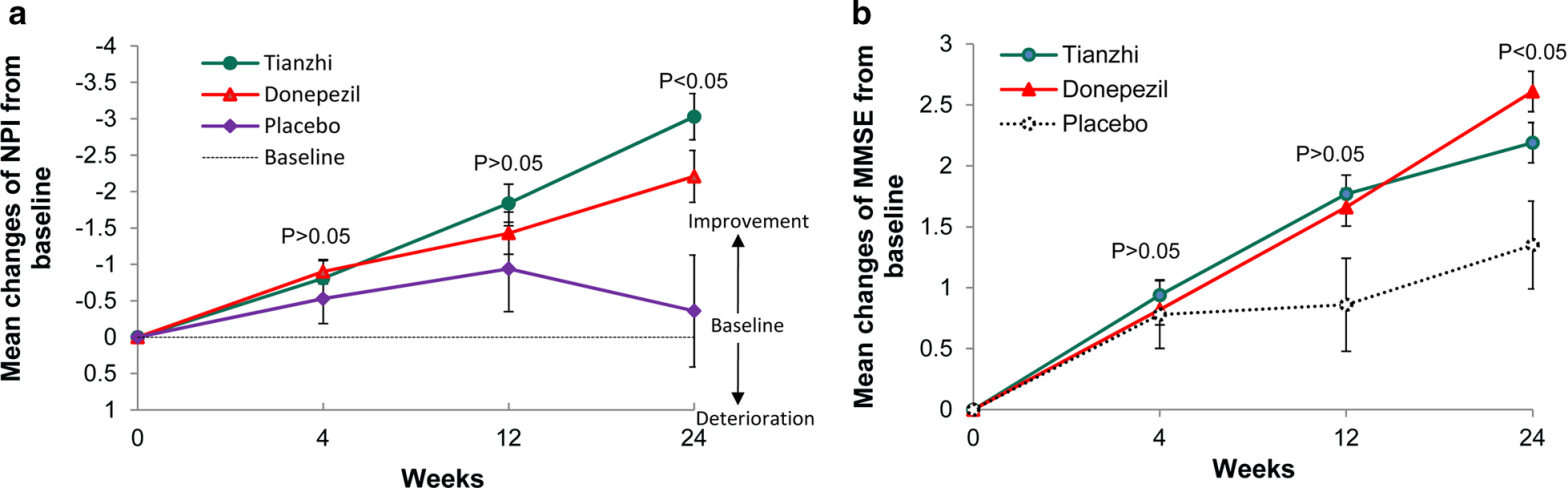
Mean change of secondary efficacy measures from baseline in three groups (ITT-LOCF analysis). **a** Mean change of NPI from baseline in three groups. **b** Mean change of MMSE from baseline in three groups. *MMSE* Mini-mental state examination, *NPI* Neuropsychiatric Inventory. p < 0.05 Tianzhi vs placebo in the mean change after 24 weeks’ treatment, p < 0.05 donepezil vs placebo in the mean change after 24 weeks’ treatment, p > 0.05 Tianzhi vs donepezil. *ITT* intent-to-treat, *LOCF* last observation carried forward, *PPS* per protocol set

At week 24, patients receiving donepezil and TZ demonstrated greater improvements from baseline levels on the NPI than placebo-treated patients in the ITT population (p = 0.019). NPI showed modest improvement from baseline in TZ (− 3.03 ± 4.84) and donepezil (− 2.21 ± 5.45) compared with placebo (− 0.36 ± 5.70) (p < 0.001 TZ compared with placebo, p = 0.013 donepezil compared with placebo) (Table 2, Fig. 3a). Although a trend of NPI improvement in TZ looked better than donepezil, there was no difference between TZ and donepezil group (p = 0.842).

#### MMSE

There were significant differences between three groups with regard to the mean changes in the MMSE scores in the ITT population (p = 0.025). In the PPS population, mean changes of the donepezil group were significantly better than that of the placebo group (p = 0.007), while there was no difference between TZ and placebo group (p = 0.07). Statistically significant benefits in favor of donepezil was apparent at week 24 (Table 2, Fig. 3b).

#### TMT

Significant improvements on the TMT-A versus placebo were observed in the donepezil group at endpoint in the ITT population. The mean changes in donepezil group were significantly higher than placebo group, TZ showed no difference compared with placebo. There was no significant difference between the three treatment groups on TMT-B. The similar trend was obtained in the PPS population.

### Numbers needed to treat

The NNTs in this study (based on improved global impression) in CIBIC-plus were 6 for TZ and 4 for donepezil.

### Safety

The proportions of patients with AE were similar among the three treatment groups, with at least one treatment-related AE experienced by 1.72% of the TZ group, 1.29% of the donepezil group, and 1.82% of the placebo groups (p = 0.78) (Table 3). One patient in the donepezil and one in placebo group suffered serious adverse events (SAE), the SAE in the donepezil group suffered acute ischemic stroke, the SAE in the placebo group suffered arrhythmia, and there were no treatment-related deaths during the study in either group. There were no clinically relevant mean changes from baseline in vital signs, or in any clinical chemistry, hematology, or urinalysis tests, in either active treatment group.

**Table 3:** Adverse events occurred in three treatment group

	Tianzhi (n = 232)	Donepezil (n = 233)	Placebo (n = 55)
	n (%)	n (%)	n (%)
Total	4 (1.72)	3 (1.29)	1 (1.82)
Diarrhea	0	1	0
Urinary tract Infection	2	0	0
Insomnia	0	0	1
Abnormal renal function	1	0	0
Arrhythmia	0	1	0
Loss of appetite	0	1	0
Bloating	1	0	0

## Discussion

In this randomized, double-blind, three arms multi-centre clinical trial, TZ and donepezil in VaD patients, demonstrated significance on both primary end points. Mild to moderate VaD patients treated with TZ or donepezil demonstrated significant benefits over placebo treated patients on measures of cognition (VADAS-cog), global impression (CIBIC-plus) and BPSD (NPI). In addition, the donepezil group showed significant improvements compared with placebo on executive function measured by the TMT-A. Both TZ and donepezil did not show benefits on ability of daily living function.

The mainly active ingredients of TZ, an approved Chinese herbal medicine by China FDA for VaD, include gastrodin, geniposide, rutin, baicalinand so on. Gastrodin improved cognitive dysfunction and decreases oxidative stress in vascular dementia rats induced by chronic ischemia [30], and the geniposide significantly alleviated neurons, apoptosis and necrosis induced by chronic cerebral hypoperfusion of cortex and hippocampus [31]. In addition, TZ could inhibit the glial cell proliferation in chronic cerebral ischemia rats [32].

Two previous donepezil studies in VaD demonstrated significant improvement in cognition and global function compared with placebo-treated patients [33, 34]. Another clinical trial of donepezil in VaD patients demonstrated slight but significant improvement on VADAS-cog, but no difference was seen on the CIBIC-Plus, suggesting that donepezil may have a greater impact on cognition than global outcomes [29]. In this study, the mean changes of donepezil and TZ group (> 4 points) in the VADAS-cog indicated that the TZ and donepezil had showed significant benefits in cognition according to previous studies which indicated an improvement of 3.3 points or more in ADAS-cog scores with anti-dementia therapy would be considered a clinically significant effect [29]. Regarding executive dysfunction, no significant treatment effects were observed for TZ, whereas a significant difference favoring donepezil was observed on the TMT-A. For activities of daily living, no difference was observed during the 24-week follow-up period. The result was consistent with a meta-analysis review on cholinesterase inhibitors, which showed significant differences in mean ADAS-cog change scores between drug and placebo, but none of the trials showed a significant effect on ADL measures [7].

NNT is defined as the average number of patients who must be exposed to an intervention to achieve the desired clinical outcome in 1 patient. We used a clinically relevant response of improvement on global function. In this study, the NNT for donepezil was 4, and TZ was 6. The NNTs in another study (based on clinically improved cognition and stable/improved global function) were 19 for donepezil [29]. The different NNTs between different studies were due to different definition of effect. The NNT in our study indicated that both donepezil and TZ showed satisfactory effect in VaD patients.

Currently, a meta-analysis showed that cholinesterase inhibitors had beneficial effects on reducing BPSD with a weighted mean difference of − 1.38 neuropsychiatry inventory point (95% CI − 2.30, − 0.46) with mild to severe AD compared with placebo [35], but most of studies was conducted in AD patients. In this large-scale VaD trial, TZ and donepezil showed modest therapeutic effects for BPSD, with a mean change of − 3.03 for TZ, − 2.21 for donepezil.

In this study, 58.19% patients showed improvement measured by CIBIC-plus in the placebo group, and the 73.71% in the TZ group, 79.72% in the Donepezil group. The placebo effect in the placebo group was consistent to the previous group, which was 52% [36].

In this study, about 70% VaD patients used concomitant medication, the proportion of patients with AE and SAE was similar among the three treatment groups, and the SAE did not lead to discontinuation from the study. And the results indicated that both TZ and donepezil were well tolerant.

There were some limitations in this study. Firstly, the ratio of test group to placebo group was 4: 1, and the patients was relative fewer in placebo; secondly, the placebo effect was higher in the placebo group. Further studies are needed to exclude subjects with particularly high placebo effects during the washout period.

## Conclusion

In summary, this large-scale, randomized, double blind, three-arms, placebo controlled trial demonstrated that, compared with placebo, TZ showed the same benefits as donepezil in terms of cognition, global impression for patients with mild to moderate VaD. Donepezil and TZ also performed potential benefits in BPSD with good tolerability.

## Data Availability

The datasets used and analysed during the current study are available from the corresponding author on reasonable request.

## Abbreviations

AD: Alzheimer’s disease
AE: Adverse events
ADL: Activity of daily living scale
BPSD: Behavioral and psychological symptoms of dementia
CIBIC-plus: Clinician’s Interview-based Impression of Change-plus caregiver information
CFDA: China Food and Drug Administration
CDT: Clock drawing test
FAS: Full analysis set
HIS: Hachinski ischemic scale
LOCF: Last observation carried forward
ITT: Intent-to-treat population
MMSE: Mini-mental state examination
NNTs: Needed to treat
NINDS-AIREN: National Institute of Neurological Disorders and Stroke–Association Internationale pour la Recherche et l’Enseignement en Neurosciences
NPI: Neuropsychiatric Inventory
PPS: Per protocol set
RCT: Randomized clinical trial
TMT: Trail making test
TZ: Tianzhi granule
VaD: Vascular dementia
VADAS-cog: Vascular Dementia Assessment Scale-cognitive subscale
